# A H2S-activated NIR-II imaging probe for precise diagnosis and pathological evaluation of colorectal tumor

**DOI:** 10.7150/thno.103999

**Published:** 2025-01-01

**Authors:** Yu Ji, Qinxian Huang, Qian Jia, Haohao Yan, Yajing Chi, Yuanyuan Jia, Chaoqiang Qiao, Yanbin Feng, Zuo Yang, Ruili Zhang, Zhongliang Wang

**Affiliations:** 1 Lab of Molecular Imaging and Translational Medicine (MITM), Engineering Research Center of Molecular and Neuro Imaging, Ministry of Education, School of Life Science and Technology, Xidian University & International Joint Research Center for Advanced Medical Imaging and Intelligent Diagnosis and Treatment, Xi'an, Shaanxi, 710126, P. R. China.; 2 Guangzhou Institute of Technology, Xidian University, Guangzhou, GuangDong, 510000, P. R. China.; 3 Department of Neurogastroenterology and Pelvic Floor Surgery, Hospital of Gastroenterology, Xi'an International Medical Center Hospital, Xi'an, Shaanxi, 710100, P. R. China.

**Keywords:** hydrogen sulfide, NIR-II imaging, molecular probe, tumor imaging, colorectal cancer

## Abstract

**Rationale:** The quick and accurate detection of colorectal cancer (CRC) is essential for improving the treatment efficacy and patient survival, which nevertheless remains challenging due to low specificity and sensitivity of current CRC diagnostic approaches. Therefore, providing a robust solution for real-time and accurate tumor delineation is highly desirable.

**Methods:** We report a novel polyacrylic acid-mediated strategy to develop the endogenous hydrogen sulfide (H_2_S)-activated NIR-II probe DCNP@PB for specific visualization of CRC and image-guided tumor surgery. The stability, biocompatibility, H_2_S-responsiveness, and NIR-II imaging capability were evaluated *in vitro* and *in vivo*. Human CRC tissues were used to evaluated the performance of the DCNP@PB.

**Results:** By exploiting the effective inner filter effect (IFE) and fluorescence resonance energy transfer (FRET) between DCNPs and PB, luminescence of DCNP@PB can be rapidly switched ON in response to H_2_S in colorectal tumor, affording high tumor-to-background ratio (TBR). Notably, H_2_S-responsive range of DCNP@PB well matches the H_2_S concentration at tumor site, thereby minimizing nonspecific activation by other sulfur-containing substances in a complicated biological environment. Such accurate H_2_S responsiveness not only benefits the differentiation between tumor and normal tissues in the mouse model, but also clearly delineates the cancerous boundaries in human tissues specimens.

**Conclusion:** This work presents not only a promising example of H_2_S-activated NIR-II optical probe that could be intravenously injected for *in vivo* applications to afford reliable information and quick feedback, but also an effective strategy to design the activatable imaging probes for precise tumor diagnosis and intraoperative decision support.

## Introduction

Colorectal cancer (CRC) is the third most common cancer diagnosis and the second leading cause of cancer death in the world 1. Over 1,918,030 new colorectal cancer cases and 609,360 deaths were estimated to occur in 2022, accounting for 10% of all cancer cases and deaths worldwide 2. Colorectal cancer survival largely depends on the stage at diagnosis, with later-stage diagnosis having poorer survival, which highlights importance of early detection and accurate diagnosis 3-6. Although several screening methods can help detect colorectal cancer, including colonoscopy, biopsy, stool sample testing, biomarker testing of the tumor, and computed tomography (CT) scan of the colon, they still suffer from certain drawbacks such as invasive testing way, low specificity and sensitivity and potential radiation risks 7-9.

Optical imaging, as a fast and real-time feedback technique, has attracted increasing attention in biomedical fields owing to its high sensitivity and selectivity, noninvasiveness, and radiation-free feature 10-13. Especially, the advent of imaging in the second near-infrared window (NIR-II, 1000-1700 nm) has further accelerated *in vivo* applications of optical imaging 14-20. Compared to conventional optical imaging approaches at shorter emission wavelengths, NIR-II imaging can significantly reduce the tissue autofluorescence, photon absorption and scattering, offering superior signal-to-background ratio and improved spatial resolution 21-23. Indeed, the past few years have witnessed the rapid development of optical probes with emission in the NIR-II region, such as single-walled carbon nanotubes (SWCNTs) 24-25, quantum dots (QDs) 26-27, rare-earth (RE)-doped nanoparticles (RENPs) 28-30, and organic molecules-based probes 31-32. Despite great progress of applying NIR-II probes for tumor imaging and imaging-guided surgery, there is still a challenge to customize a highly specific and sensitive NIR-II probe to improve the diagnostic accuracy of CRC. The utilization of RENPs-based NIR-II probes to achieve this goal has been even less explored.

Hydrogen sulfide (H_2_S), as the third gaseous transmitter, has been proven to play important roles in various physiological and pathophysiological processes 33-34. The increased production of H_2_S, generated by overexpressed cystathionine-β-synthase (CBS) in colorectal cancer cells contributes to cancer cell proliferation, migration, and invasion, which makes H_2_S a promising chemical target for identification of CRC 35-37. Therefore, the H_2_S-responsive probes have been recently fabricated and used to detect CRC. However, most of the H_2_S-responsive probes are administrated intratumorally, which may not reflect the complexity of *in vivo* situation 38. Additionally, although emphasis is often placed on the detection limit of probes toward H_2_S, the detection window is more crucial to guarantee detection specificity. The probe whose H_2_S-responsive range matches the H_2_S concentration at tumor site helps to avoid the interference from other diseases or organs that also contain H_2_S or other sulfur species, holding great potential for *in vivo* imaging of CRC 39. Therefore, to develop a H_2_S-activatable NIR-II optical probe with a good stability and highly matched responsive-range is extremely desirable for sensitive and accurate CRC detection.

**Scheme 1:**
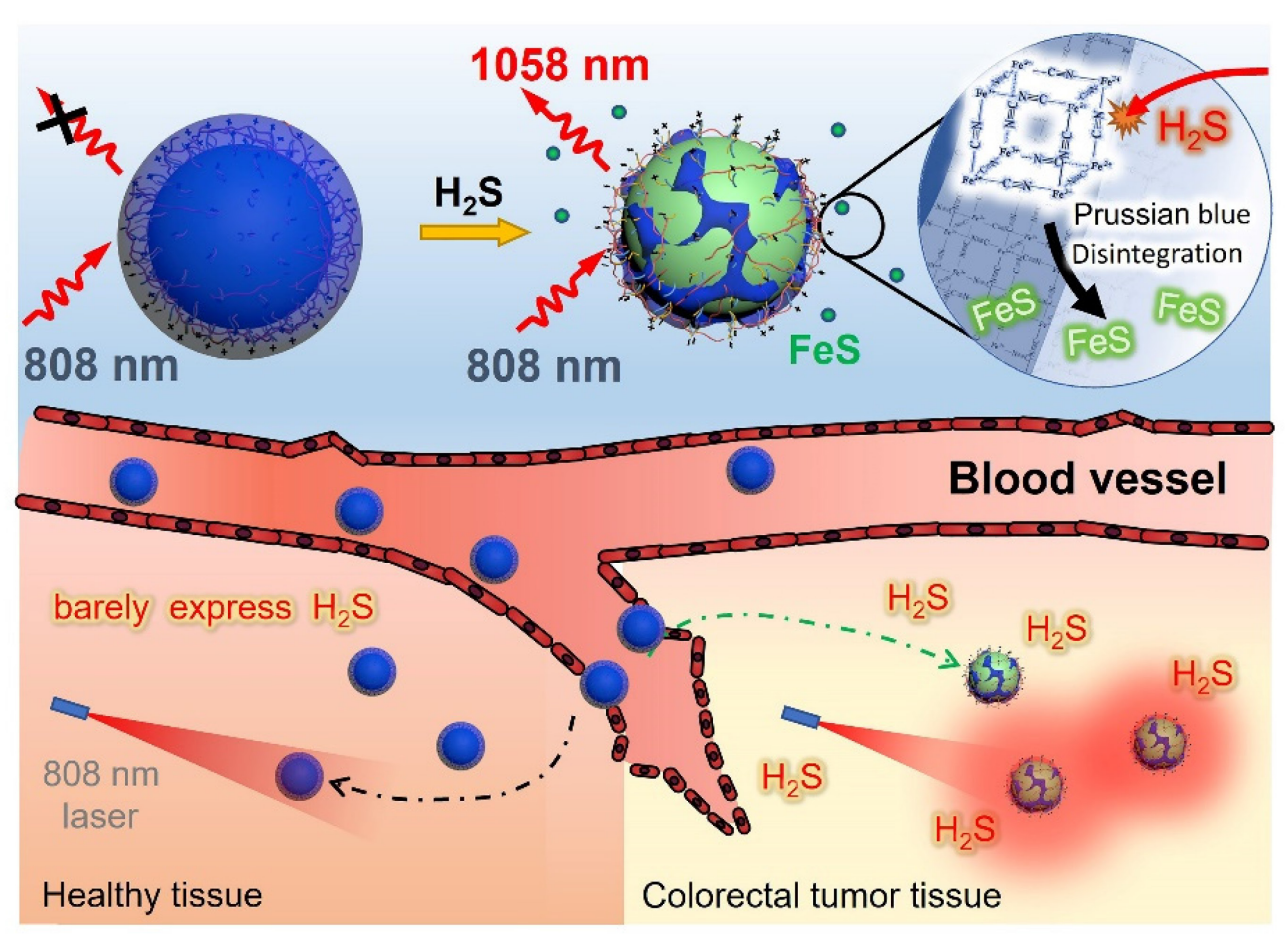
Schematic illustrations of an endogenous H_2_S-activatable NIR-II nanoprobe DCNP@PB for highly specific imaging of colon tumor.

Herein, we proposed a novel polyacrylic acid (PAA)-mediated strategy to fabricate an endogenous H_2_S-activated NIR-II probe based on Nd^3+^-doped downconversion nanoparticles (DCNPs) coated with Prussian Blue (PB) layer (termed DCNP@PB) for sensitive and highly specific detection of CRC (Scheme 1). Besides serving as a H_2_S-responsive motif, PB plays a crucial role in regulating the emission properties of DCNP@PB. After encapsulation by PB, the luminescence of DCNPs is greatly quenched via both inner filter effect (IFE) and fluorescence resonance energy transfer (FRET) due to spectral overlap between absorption of PB and excitation/emission spectra of DCNPs. However, once at tumor site, the Fe(III) in PB reacts with highly expressed H_2_S and causes the decomposition of PB layer, leading to a significant decrease in PB absorbance. As a result, the luminescence of DCNPs can be recovered to illuminate the colorectal tumor with high sensitivity and specificity. Most importantly, H_2_S-responsive range of DCNP@PB well matches the H_2_S concentration at tumor site (0.3-3.4 mmol L^-1^) 40, allowing the specific detection of colorectal tumor in complicated physiological environment. With these favorable features, intravenously administrated DCNP@PB not only exhibits superior capability for *in vivo* CRC imaging in mouse models, but also well defines margins of the tumor lesions with high accuracy in human colorectal tissues.

## Materials and methods

### Reagents and instrumentation

All chemical reagents, if not specified, were purchased from Alfa Aesar or Sigma-Aldrich and used without purification. Deionized water (DI water) was used in all experiments. Murine mammary carcinoma cell line 4T1, HCT116, Luciferase-expressing HCT116 were obtained from Shanghai Zhong Qiao Xin Zhou Biotechnology Co., Ltd. (Shanghai, China). Penicillin streptomycin, RPMI 1640 Medium, bovine serum (FBS) and 0.25% (w/v) trypsin solution were purchased from Gibco Life Technologies (AG, Switzerland). Phosphate-Buffered Saline (PBS) was purchased from OpticsPlanet, Inc. (USA). BALB/c nude female mice (6-7 weeks old and weighing 18-22 g) were supplied by the Animal Center of the Fourth Military Medical University (Xi'an, China). Animal protocols related to this study were reviewed and approved by the Institutional Animal Care and Use Committee of the Fourth Military Medical University (approval number: 20220312). The tissues taken from the patients related to this study were reviewed and approved by the Medical Research Ethics Committee of Xi 'an International Medical Center Hospital (approval number: GJYX-KY-2024-023). All participants provided written informed consent before study enrollment. Transmission electron microscopy (TEM) images were obtained using a JEM-2100F electron microscope. NIR-II live animal imaging system (Series II 900/1700-H, China) and home-built small animal NIR-II imaging system by InGaAs-NIRvana640LN camera were applied for *in vivo* imaging. NIR-II microscopy platform (InGaAs CCD camera equipped with a microscope objective lens) was self-built 41.

### Synthesis of NaGdF_4_: Nd nanoparticles

In a typical synthetic procedure of NaGdF_4_:5%Nd, 1 mmol of CF_3_COONa, 0.95 mmol of Gd(CF_3_COO)_3_, 0.05 mmol of Nd(CF_3_COO)_3_ were mixed with oleic acid (10 mmol), oleylamine (10 mmol) and 1-octadecene (20 mmol) in a two-neck reaction flask. The slurry mixture was heated to 120 °C under vacuum for 30 min to remove water and oxygen. Then the solution was heated to 300 °C with a rate of 10 °C/min under dry argon flow, and then maintained at 300 °C for 60 min with stirring. After cooling to room temperature, an excess amount of ethanol was poured into the solution. The resultant nanoparticles were centrifuged at 5,000 r.p.m. for 20 min. After centrifugal washing with hexane/ethanol, NaGdF_4_:5%Nd nanoparticles were re-dispersed in 5 mL of hexane for further coating.

### Synthesis of NaGdF_4_: Nd@NaGdF_4_ nanoparticles

Typically, 5.0 mL of as-prepared NaGdF_4_:5%Nd solution, 1 mmol of CF_3_COONa, 1 mmol of Gd(CF_3_COO)_3_ were mixed with oleic acid (20 mmol) and 1-octadecene (20 mmol) in a two-neck reaction flask. Then the mixture was heated to 120 °C under vacuum for 30 min to remove hexane and water. The flask was then heated to 305 °C for 75 min with stirring. After cooling to room temperature, an excess amount of ethanol was poured into the solution. The resultant nanoparticles were centrifuged at 5,000 r.p.m. for 20 min. After centrifugal washing with hexane/ethanol, NaGdF4:5%Nd@NaGdF4 nanoparticles were re-dispersed in 5 mL of hexane for further coating.

### Synthesis of NaGdF_4_: Nd@NaGdF_4_@NaGdF_4_ nanoparticles

The synthetic procedure of NaGdF_4_:5%Nd@NaGdF_4_@NaGdF_4_ was the same as that used to synthesize NaGdF_4_:5%Nd@NaGdF_4_ nanoparticles, except that 5 mL of as-prepared NaGdF_4_:5%Nd@NaGdF_4_ solution, 1 mmol of CF_3_COONa, 1 mmol of Nd(CF_3_COO)_3_ were mixed with oleic acid (20 mmol) and 1-octadecene (20 mmol) in a two-neck reaction flask. The as-synthesized NaGdF_4_:5%Nd@NaGdF_4_@NaGdF_4_ nanoparticles were re-dispersed in 10 mL of chloroform for further modification.

### Synthesis of NaGdF_4_: Nd@NaGdF_4_@NaGdF_4_@PVP nanoparticles

2 g of polyvinyl pyrrolidone (PVP) was dissolved in 20 mL of DI water in 5 mL flask, and then 1 mL NaGdF_4_:5%Nd@NaGdF_4_@NaGdF_4_ in chloroform was added dropwise, followed by 6 h of stirring at room temperature. Subsequently, after centrifugation at 14,000 r.p.m for 1 h, the excess surfactant was removed to obtain hydrophilic nanoparticles NaGdF_4_:5%Nd@NaGdF_4_@NaGdF_4_@PVP, which were dissolved in 10 mL of DI water for later use coating.

### Preparation of DCNP@PB probe (NaGdF_4_: Nd@NaGdF_4_@NaGdF_4_@PVP@PB)

4 mg Polyacrylic acid (PAA, MW: 1800) added into 10 mL of NaGdF_4_:5%Nd@NaGdF_4_@NaGdF_4_@PVP, followed by 1 h of stirring at room temperature. Then 2 mg of FeCl_3_ dissolved in 1 mL of DI water was added to solution under magnetic stirring, followed by 1h of stirring at room temperature. Afterwards, 30 mL isopropanol was added to the above mixed suspension under stirring for 30 min. Then 5 mg K₄[Fe (CN)_6_] aqueous solution was added to the above mixed suspension, followed by 12 h of stirring at room temperature. Subsequently, after centrifugation at 10000 r.p.m. for 10 min, the excess surfactant was removed to obtain hydrophilic nanoparticles NaGdF_4_:5%Nd@NaGdF_4_@NaGdF_4_@PVP@PB probe, which were dissolved in 1 mL of DI water.

### Preparation of NdNP@PAA-mPEG probe

4 mg Polyacrylic acid (PAA, MW: 1800) added into 10 mL of NaGdF_4_:5%Nd@NaGdF_4_@NaGdF_4_@PVP, followed by 1 h of stirring at room temperature to obtain NdNP@PAA solution. Then 4 mg mPEG-NH_2_ (5k) mixed in 3 mL MES solution is added to above NdNP@PAA solution. 8 mg EDC is added then. The solution is shakily reacted for 3 hours. Then 20 mg Tris-base and 10 mg EDC is added into above solution. The solution is shakily reacted for another 3 hours. The solution is centrifuged at 4500 r.p.m. for 30 minutes to get rid of potential large floccules. Then the supernate is washed against DI water by 100 K centrifugal filter for 3 times. The final ErNPs-mixPEG are dispersed in 800 μL MES solution.

### Synthesis of ErNP, ErNP@PVP and ErNP@PB

The ErNP nanoparticle was synthesized by the literature method 42. The synthetic procedure of ErNP@PVP and ErNP@PB was the same as that used to synthesize NaGdF_4_@PVP and DCNP@PB nanoparticles.

### Detection of H_2_S *in vitro*

The H_2_S standard solution was prepared by adjusting the pH of freshly formulated NaHS solution to neutral (pH = 7.4) with diluted hydrochloric acid solution. All H_2_S standard solutions and DCNP@PB nanoprobes suspension were used immediately after formulation. To set up the standard curve for H_2_S detection, 1 mL DCNP@PB nanoprobes solution (1 mg mL^-1^) was added into 1 mL H_2_S standard solutions with concentrations from 0 μM to 800 μM, respectively. After incubated for 10 min, the UV-vis-NIR absorbance spectra and NIR spectra of all samples were determined, respectively.

The Signal-to-Noise Ratio Method is used in the calculation of LOD (Limit of Detection):

LOD=3σ/S

where σ is the standard deviation of the background noise and S is the slope of the calibration curve (response versus concentration).

The same method to demonstrate the selectivity of DCNP@PB nanoprobes, including common biosulfurs (reduced glutathione (GSH), cysteine (Cys), glutamic acid (Glu), SO_3_^2-^), essential/non-essential amino acids, reactive oxygen species (Vitamin C (VC), HClO, H_2_O_2_, and ONOO^-^), cations (K^+^, Na^+^, Ca^2+^, Mg^2+^, Mn^2+^, Zn^2+^, and NH^4+^), anions (Cl^-^, PO_4_^3-^), dopamine, bovine serum albumin (BSA). The concentration of studied biomolecules and ions was 10 mM.

To demonstrate the speed of responsive of DCNP@PB nanoprobes, 1 mL DCNP@PB nanoprobes suspension (10 mg mL^-1^) were monitored dynamically per 6 s by UV-vis-NIR absorbance spectra. Time to 2 min, 1 mL H_2_S standard solution (600, 300, 0 μM) was added rapidly. Continuous monitoring to 5 min, UV-vis-NIR absorbance spectra of all samples were determined.

### Stability of DCNP@PB

The intensity at 1058 nm of DCNP@PB nanoprobes in various biological solutions (DI water, PBS (pH 7.4, pH 6.5, saline, 0.35 mg mL^-1^) were recorded by and NIR spectra within 120 min. The DLS of DCNP@PB after various storage time points were determined after voticity for 2 h.

### Cell lines culture

Murine mammary carcinoma cell line 4T1, human colon cancer cell line HCT116 and luciferase HCT116 (HCT116-luc), were cultured in Dulbecco's modified Eagle's medium (DMEM) with 10% fetal bovine serum (FBS) at 37 °C under 5% CO_2_.

### Cytotoxicity test *in vitro*

The cell viability was measured using conventional MTT assay. The used cell lines were human colon cancer cell line HCT116. Typically, cells were respectively seeded at a density of 5000 cells per well in a 96-well plate. After 12 h of incubation, fresh cell culture medium containing various concentrations (0, 0.5, 1, 2, 4, 6, 8 and 10 mg mL^-1^) of DCNP@PB was added for further incubation of 24 h. To determine the cell viability, cells were washed with PBS for three times and incubated with MTT (5 mg mL^-1^) solution for another 4 h, followed by 150 μL of DMSO was added to each and optical absorption measurement at 492 nm to calculate cell viability.

### Cell imaging

2×10^4^ HCT116 cells were pretreated with PBS, aminooxyacetic acid AOAA (2 mM) for 24 h, 2×10^4^ 4T1 cells were pretreated with PBS for 24 h. Group1 cells with various pretreatments were then incubated with DCNP@PB nanoprobes suspension (1 mg mL^-1^) for 4 h, Group2 cells with various pretreatments were then incubated with DCNP@PB nanoprobes suspension (1 mg mL^-1^) and NaHS (200 μM) for 4 h, washed with PBS several time. The cell imaging was taken by NIR-II live animal imaging systemexicted by 808 nm laser (100 mW cm^-2^). For calibration experiments, the cultured cells were counted using an automated cell counter. Cell suspensions with different cell concentrations were added to a row of wells. Following this, 10 μL of 1 mg mL^-1^ D-luciferin-K^+^ salt bioluminescent substrate in PBS is added to each well. After a 3 min reaction time, NIR-II imaging *ex vivo* bioluminescence imaging is performed.

### Animal models construction

To establish colon cancer and breast cancer mice model, approximately 5×10^5^ HCT116 and 4T1 cells in 5 μL phosphate buffered saline were intracranially injected into the right hindlimb of the BALB/c mice subcutaneously.

### *In vivo* imaging

Before imaging, the hair of all the mice was removed by using hair clipper and depilatory cream. The mice were intravenously injected with 100 µL (10 mg mL^-1^) probes. 50 µL 10 mg mL^-1^ NaHS solution was injected subcutaneously around the tumor during the 4T1 tumor imaging. Human *ex vivo* tissues were immersed in a 10 mg mL^-1^ probe solution for 6 hours. Fluorescence images were acquired by Series II 900/1700-H. An 808 nm laser diode was decided to be the excitation laser for both contrast agents at a power density of 400 mW cm^-2^ (lower than the safe exposure limit of 1.0 W cm^-2^ determined by the International Commission on Nonionizing Radiation Protection). A 1000 nm long-pass filter were applied to collect the emission of the probes. Exposure time was 50 ms for all images.

### Statistical analysis

Data analysis was conducted using Origin 9, GraphPad Prism 8, LivingImage and ImageJ v.2.9.0. All data, presented as mean ± s.d., were calculated using Origin 9 or GraphPad Prism 8. Statistics were performed on SPSS17.0 statistical analysis software. All the error bars indicated mean ± standard deviation. Statistical analysis was performed using Student's t test (two-tailed). Statistical significance was established as indicated * *P* < 0.05; ** *P* < 0.01 and *** *P* < 0.001.

## Results and Discussion

### Synthesis and characterization of DCNP@PB nanoprobe

**Figure 1:**
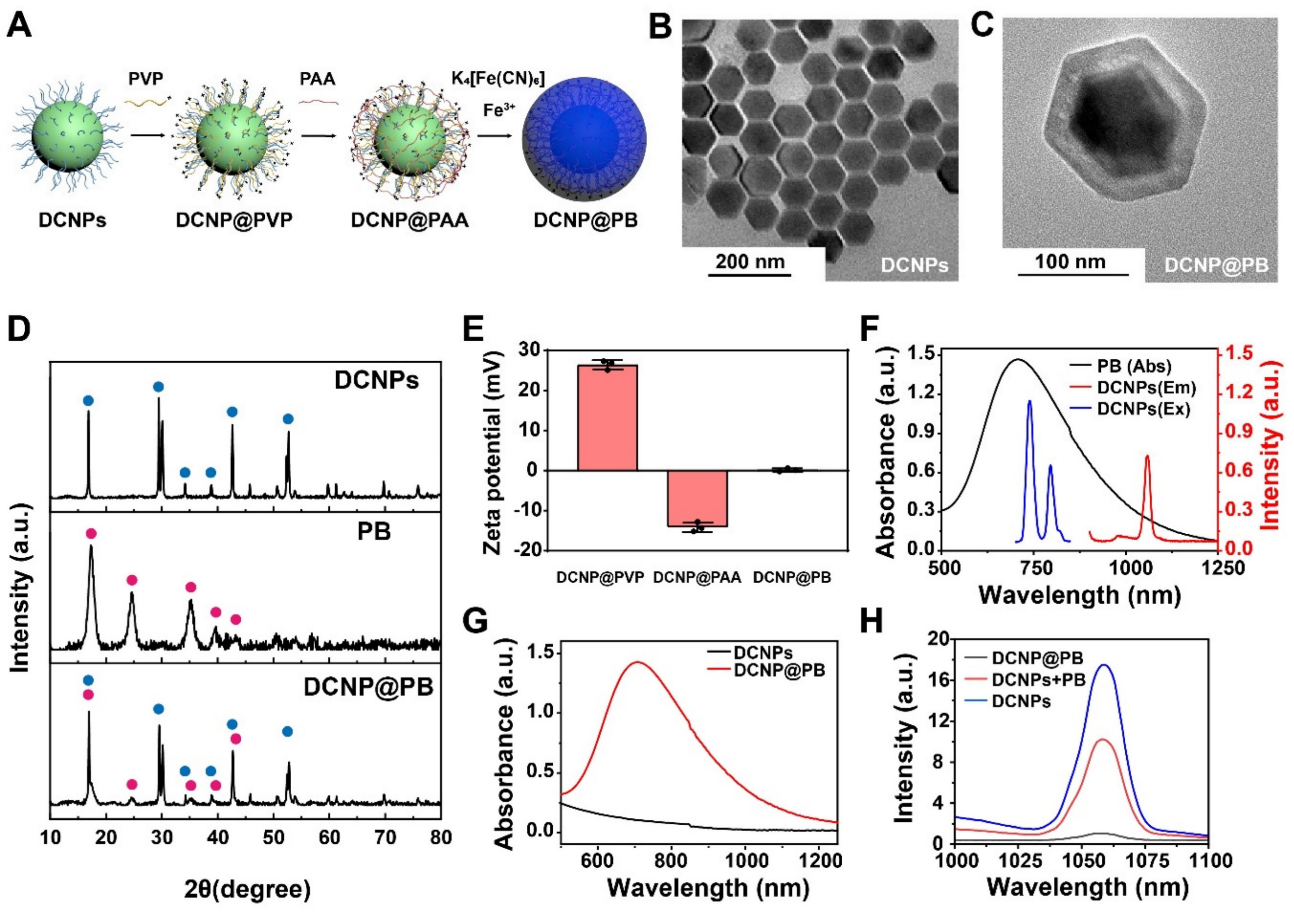
Characterization of the DCNP@PB nanoprobes. (A) Schematic illustrations of synthesis of DCNP@PB. (B) TEM image of DCNPs. (C) TEM image of DCNP@PB. (D) X-ray diffraction spectra of DCNPs, PB and DCNP@PB. (E) Zeta potential of DCNP@PVP, DCNP@PAA and DCNP@PB. (F) Absorption spectrum of PB, excitation and emission spectra of DCNPs. (G) Absorption spectra of DCNPs and DCNP@PB. (H) NIR-II emission spectra of DCNPs, DCNPs in PB solution and DCNP@PB.

DCNP@PB probe composed of Nd^3+^-doped downconversion nanoparticles (DCNPs) NaGdF_4_: Nd@NaGdF_4_ interior and a PB coating were synthesized using a novel PAA-mediated strategy (Figure 1A and Figure S1A-1C). Briefly, oleic acid-capped DCNPs were first modified with a water-soluble polymer, PVP through a ligand-exchange approach. PAA was then introduced onto the surface of DCNPs via an electrostatic interaction, endowing the DCNPs with negatively charged surface. Fe^3+^ was added to PAA-capped DCNPs to yield a PAA/Fe^3+^ solution, followed by adjusting pH of reaction system. Afterward, isopropanol and K_4_[Fe(CN)_6_] were added to this mixture for PB growth and mineralization to finally generate DCNP@PB. The representative TEM images in Figure 1B and C showed that as-prepared DCNPs (88.59 nm ± 2.1 nm) were homogenously encapsulated by a PB layer, resulting in particle with an average diameter of approximately 140.16 ± 1.7 nm. Further elemental mapping analysis demonstrated that the obtained DCNP@PB was uniformly composed of Gd (green), Nd (purple), and Fe (red) (Figure S1D). The successful formation of DCNP@PB was also confirmed by X-ray diffraction (XRD) patterns results, which clearly showed combined characteristic peaks of hexagonal phase of DCNPs and PB (Figure 1D). In addition, shift in zeta potential of DCNPs surface indicated the fabrication of DCNP@PB, which has a near-neutral net surface charge at physiological pH (Figure 1E).

To evaluate the luminescence behaviors of DCNP@PB, we first measured the optical spectra of DCNPs and PB, respectively. As shown in Figure 1F, excitation and emission spectra of DCNPs effectively overlapped with absorption spectra of PB, indicating the possibility of IFE or FRET. To further investigate the effects of the PB on emission of DCNP, we tracked the absorption and emission spectra of DCNPs before and after PB coating. The mixture of DCNPs with PB was used for comparison. As shown in Figure 1G, DCNP@PB displayed a typical broad absorbance band of PB extending to beyond 1200 nm with peak absorbance at 720 nm, suggesting the presence of PB shell. Meanwhile, the luminescence of DCNP@PB was almost completely quenched compared with that of pure DCNPs, which exhibited characteristic emission bands of Nd^3+^ at 1058 nm under an 808 nm laser irradiation (Figure 1H). However, when DCNPs directly mixed with PB, in which the DCNPs and PB was not too close to induce significant FRET, the emission intensity of DCNPs only decreased by half (Figure 1H). These results indicated that single IFE between DCNPs and PB is not enough to strongly quench luminescence of DCNPs and both IFE and FRET mechanisms may be responsible for substantial reduction in DCNP@PB luminescence.

To verify our assumption, we fabricated a PB coated Er^3+^-doped nanoparticles with 1525 nm emission (denoted as ErNP@PB) to diminish the spectra overlap between absorbance of PB and emission of ErNPs. In this way, the FRET could be excluded. By keeping the absorbance at 720 nm the same (Figure S2A, 2.5 mg of PB), we further compared the emission of ErNP@PB with that of mixture of ErNPs and PB. As shown in Figure S2B, luminescence of both groups declined slightly compared with that of pure ErNPs. In addition, there is almost no difference in their emission intensity (2.5 mg of PB), which indicated that the luminescence quenching of ErNP@PB can be mainly attributed to the IFE. Moreover, the emission intensity of the mixture was gradually dropped with the increasing amount of PB, further indicating the existence of IFE (Figure S2B). We also investigated the emission spectra of ErNPs with or without PB coating under 980 nm or 808 nm excitation, which would result in the different degree of spectral overlap between ErNPs and PB. As shown in Figure S2C and D, when excited by 808 nm laser, luminescence of ErNP@PB decreased more sharply than that under 980 nm excitation, indicating the existence of IFE. Taken together, it can be reasoned that the significant luminescence quenching of DCNP@PB could be attributed to both FRET and IFE between DCNPs and PB. In addition to the matched optical properties, the distance between DCNPs and PB is crucial. Benefiting from our innovative surface functionalization strategy, PB could be evenly and densely coated onto DCNPs surface to permit optimal FRET, considering that the energy donor-acceptor pair must be close enough (typically 1 - 10 nm) for effective FRET.

### *In vitro* evaluation the H_2_S response characteristics of DCNP@PB nanoprobe

**Figure 2:**
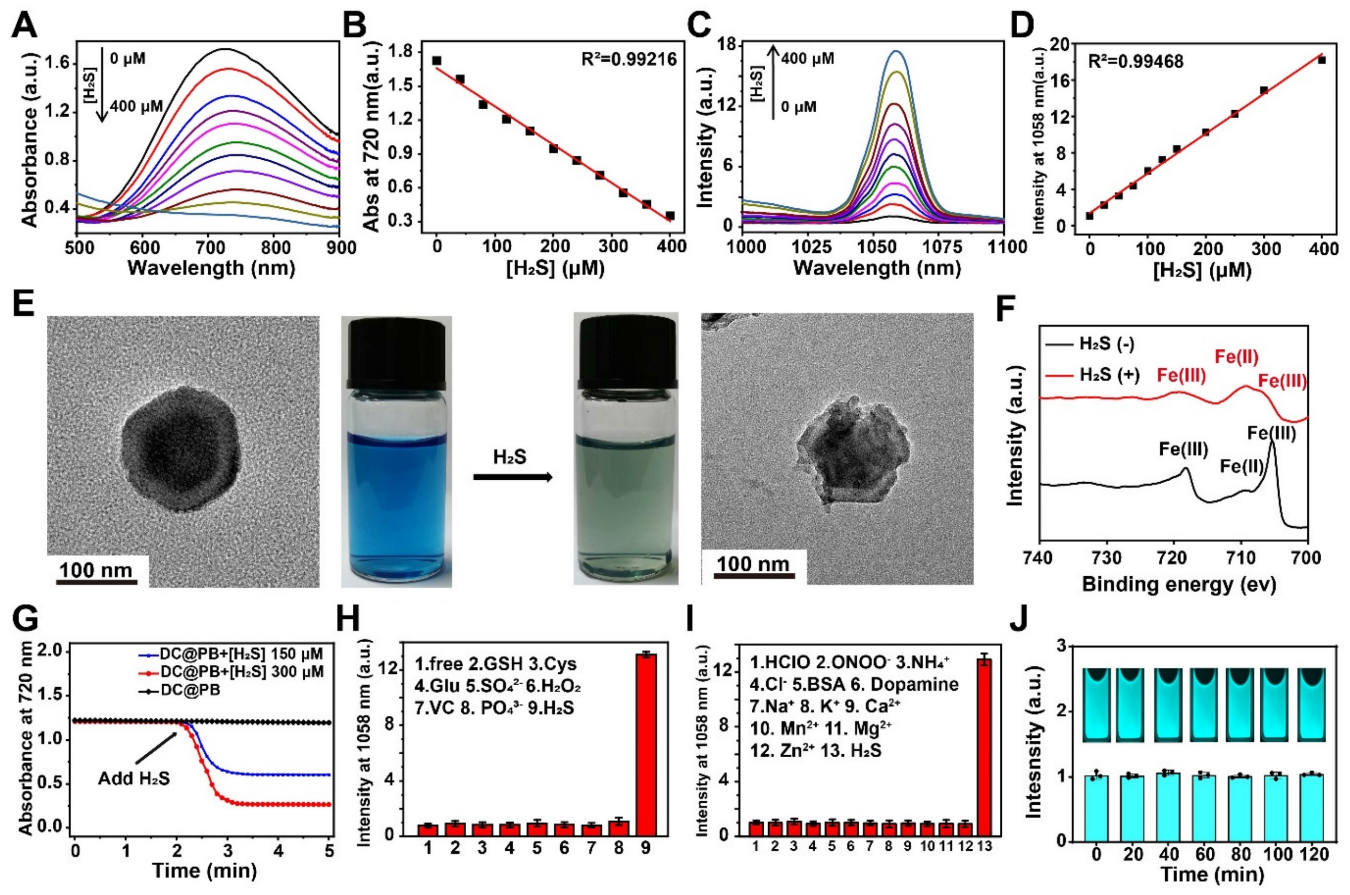
Evaluation of H_2_S response characteristics of DCNP@PB nanoprobe. (A) Absorption spectra of the DCNP@PB in H_2_S solutions with different concentration (0-400 μM). (B) The absorbance at 720 nm of DCNP@PB was plotted against the H_2_S concentration generate an empirical calibration formula with R^2^ = 0.992. (C) NIR-II emission spectra of DCNP@PB in H_2_S solutions with different concentration (0-400 μM) under excitation wavelengths of 808 nm. (D) The intensity at 1058 nm of DCNP@PB was plotted against the H_2_S concentration generate an empirical calibration formula with R^2^ = 0.995. (E) TEM images and photo of DCNP@PB solution (left) and H_2_S-responsed DCNP@PB solution (right). (F) Fe_2p_ XPS spectra of DCNP@PB with/without H_2_S addition. (G) The response time reflected by changes in the absorbance at 720 nm of DCNP@PB. (H) and (I) The normalized luminescence intensity of DCNP@PB in the presence of H_2_S or other active substances. (J) Photostability of DCNP@PB solution under continuous 808 nm laser irradiation.

To explore the H_2_S-responsive performance of DCNP@PB, the changes in absorption spectra of PB and emission spectra of DCNP@PB were monitored at different H_2_S concentration. As shown in Figure 2A and B, as the concentration of H_2_S increased from 0 to 400 µM, the absorbance of PB gradually decreased, demonstrating the decomposition of the PB shells in the presence of H_2_S. As a result, the luminescence of DCNP@PB at 1058 nm enhanced dramatically (more than 17-fold), indicating the fast activation of DCNP@PB in response to H_2_S (Figure 2C and D). This H_2_S-responsive decomposition process of the DCNP@PB could also be confirmed by the TEM results and photographs observed in Figure 2E, which clearly showed that the intact PB shell was disintegrated after addition of H_2_S along with a color change from blue to dark green. Consistently, Fe 2p X-ray photoelectron spectroscopy (XPS) spectra supported the reduction of Fe(III) and the formation of FeS (Figure 2F) in the presence of H_2_S. DCNP@PB responded to the addition of the H_2_S in less than 20 s (Figure 2G). This fast response makes DCNP@PB suitable for real-time tumor imaging *in vivo*.

Furthermore, the H_2_S selectivity of DCNP@PB was evaluated with various analytes, including other reducing biological species, such as cysteine (Cys) and glutathione (GSH) at 100 μM. The results presented in Figure 2H-I and Figure S3 clearly showed that only H_2_S could cause a dramatic change in the emission and absorption of DCNP@PB under the identical conditions, demonstrating that DCNP@PB was promising for specific detection of H_2_S in a complicated biological environment. Notably, DCNP@PB could detect H_2_S with a detection limit of 10 µM according to the linear correlation between the NIR-II luminescence intensities and NaHS concentrations of 0-400 µM. This detection window just fell in the concentration range of H_2_S in CRC, implying that DCNP@PB may be able to specifically distinguish the colorectal cancer from other organs or disease based on the difference in concentration of H_2_S.

Furthermore, DCNP@PB showed great photostability under 808 nm laser irradiation (Figure 2J), which is favorable for intraoperative imaging. The stability of DCNP@PB in water, PBS buffer, DMEM, RPMI 1640 Medium, and 10% fetal bovine serum complete medium were tested by measuring the hydrodynamic size of DCNP@PB. As shown in Figure S4A, the size of DCNP@PB remained nearly unchanged over 12 h and exhibited excellent colloidal stability in physiological media. Additionally, the emission intensity of DCNP@PB at 1058 nm has barely changed for 2 h even at acidic tumor extracellular pH (6.5), indicating that DCNP@PB is suitable for imaging of CRC with stable NIR-II luminescence (Figure S4B). In addition, the cytotoxicity of DCNP@PB towards colorectal cancer cells HCT116 as assessed. As shown in Figure S5, no significant cytotoxicity was observed at various concentrations (up to 10 mg mL^-1^), suggesting excellent biocompatibility of DCNP@PB.

**Figure 3:**
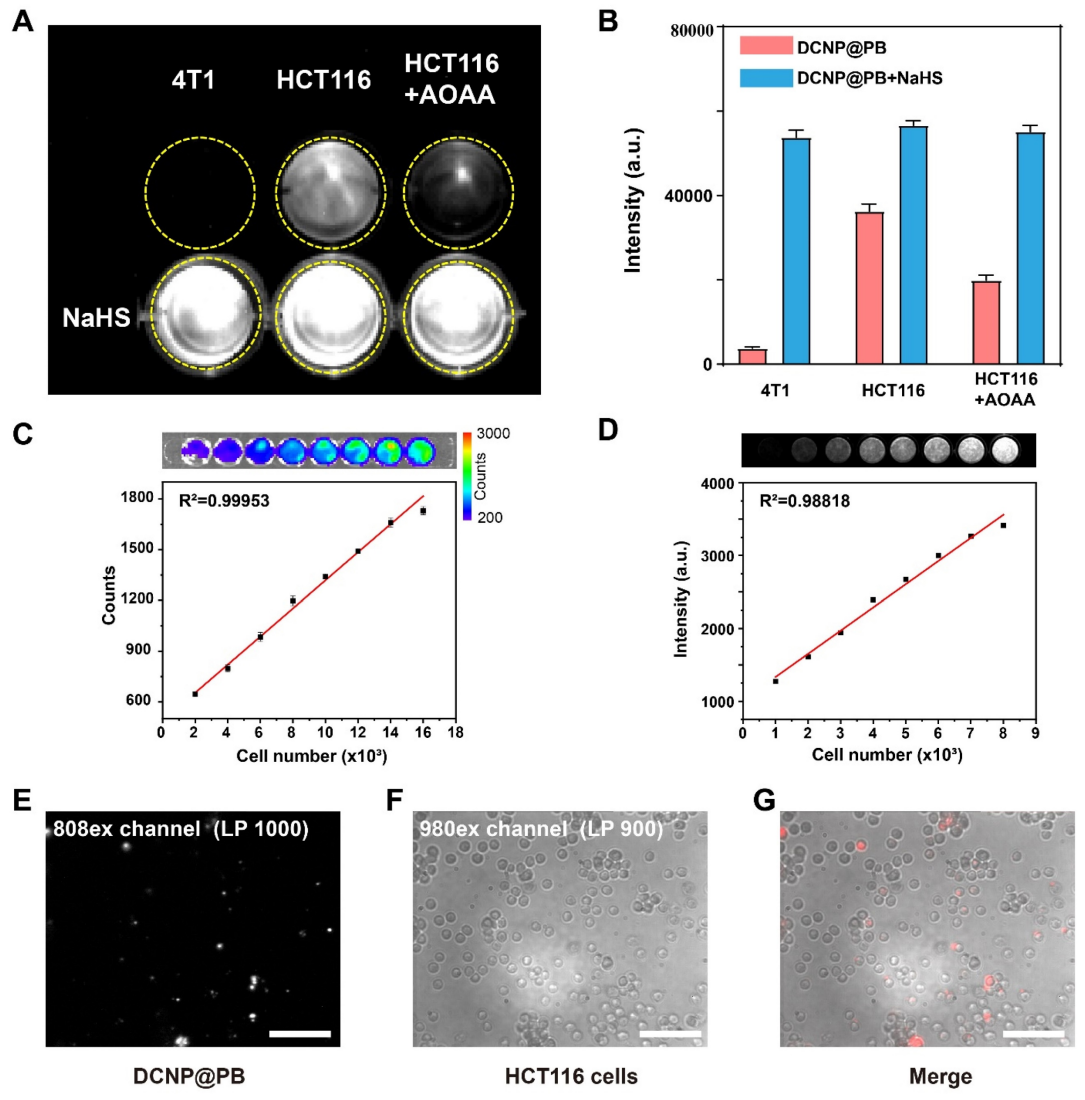
H_2_S-responsive performance of DCNP@PB on cells. (A) NIR-II imaging of 4T1 cells and HCT116 cells incubated with DCNP@PB after treatment with AOAA or NaHS. (B) Quantitative luminescence intensity of (A). (C) *Ex vivo* bioluminescence imaging of luciferase-expressing HCT116 cells in PBS solutions with different cell numbers (upper); and the corresponding luminescence intensity counts were plotted against the cell numbers (lower). (D) NIR-II imaging of luciferase-expressing HCT116 cells incubated with DCNP@PB in PBS solutions with different cell numbers (upper); and the corresponding intensity were plotted against the cell numbers (lower). (E) 20x NIR-II micro-imaging of HCT116 cells incubated with DCNP@PB in 808-EX channel (long pass 1000 nm), (F) 980-EX channel (long pass 900 nm) and (G) merging image of two channels. Scale bar: 100 μm.

To further verify whether the DCNP@PB could target H_2_S-expressed colorectal cancer and initiate DCNP@PB emission effectively, H_2_S-rich HCT116 human colorectal cancer cells and H_2_S-deficient 4T1 breast cancer cells were used to evaluate the cell imaging performance of DCNP@PB. As shown in Figure 3A, HCT116 cells incubated with DCNP@PB afforded bright NIR-II luminescence under excitation of 808 nm laser, while same treatment with 4T1 cells did not elicit detectable signals. To confirm that the luminescence of DCNP@PB was indeed activated by endogenous H_2_S in HCT116 cells, AOAA, a frequently used CBS inhibitor, was utilized to suppress the synthesis of H_2_S in cells. As depicted in Figure 3A and B, pretreatment of HCT116 cells with AOAA did result in a substantial decrease in DCNP@PB luminescence, indicative of the involvement of H_2_S in DCNP@PB activation. In contrast, when NaHS was introduced to up-regulate H_2_S content in cells before incubated with DCNP@PB, enhanced luminescence signals can be observed for both 4T1 and HCT116 cells. Moreover, the luminescence intensity of DCNP@PB in HCT116 cells increased linearly with the number of cells (Figure 3C and D). All these results demonstrated that the NIR-II luminescence recovery of DCNP@PB probe is closely related with H_2_S contents in cells, which enables imaging of H_2_S-rich HCT116 cells with high sensitivity and specificity. Furthermore, HCT116 cells incubated with DCNP@PB showed clear and independent luminescence (Figure 3E-G), indicating that DCNP@PB can be internalized by HCT116 cells and activated by H_2_S effectively.

### Sensitivity and specificity of DCNP@PB for *in vivo* tumor imaging

**Figure 4:**
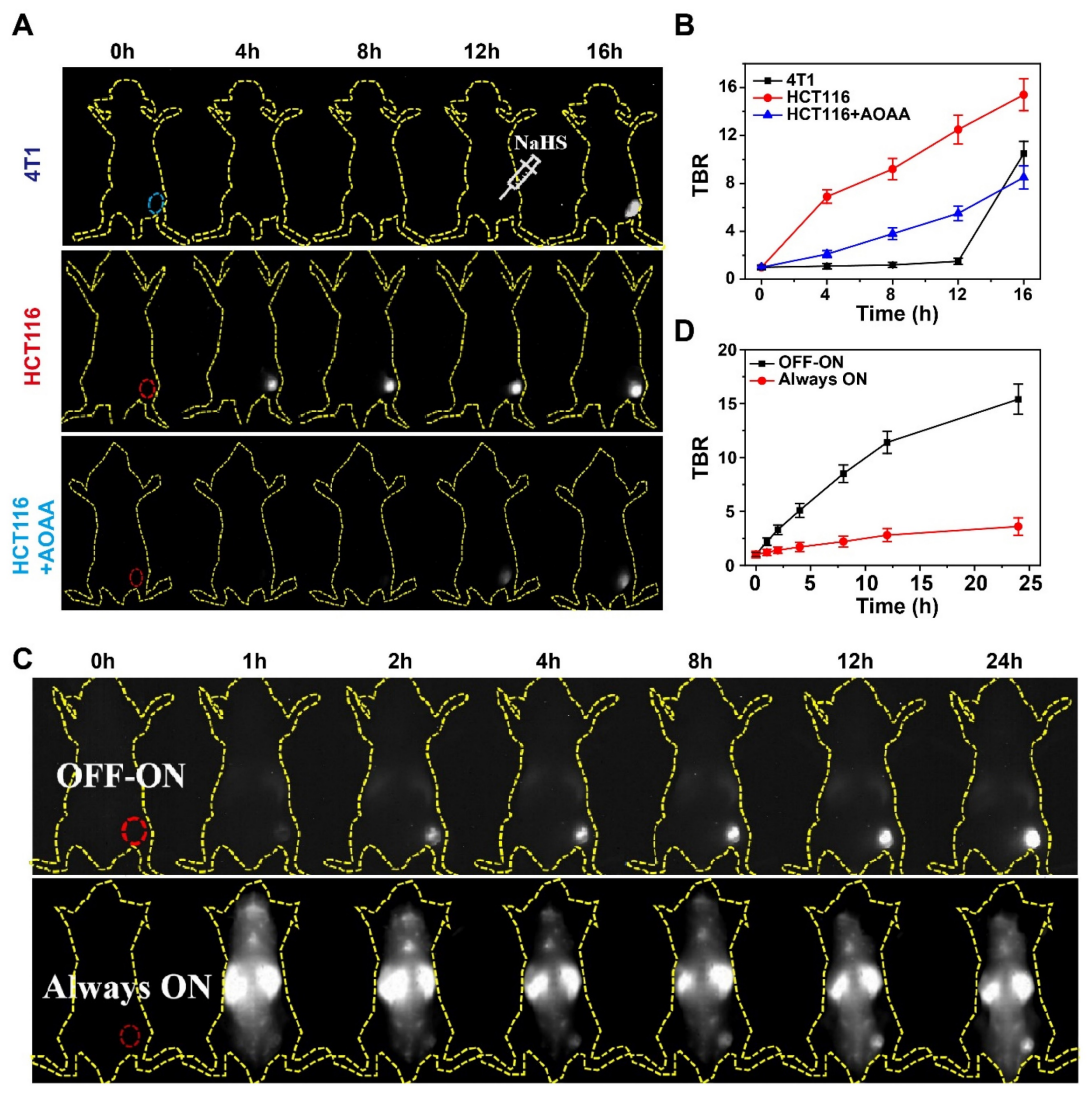
The specificity of DCNP@PB for tumor extracellular H_2_S-responsive imaging. (A) Time-dependent NIR-II luminescence imaging of mice bearing 4T1 and HCT116 cells tumor after intravenous injection of DCNP@PB under different conditions. (B) TBR as a function of time from A. (C) Time-dependent NIR-II luminescence imaging of mice bearing HCT116 cells tumor after intravenous injection of DCNP@PB (upper) and NdNP@PAA-mPEG (lower), respectively. (D) TBR as a function of time from (C).

Encouraged by the impressive H_2_S-responsiveness of DCNP@PB *in vitro*, we further evaluated the capability of DCNP@PB to identify the colorectal cancer *in vivo*. Toward this goal, DCNP@PB was first intratumorally injected into HCT116 or 4T1 tumor-bearing mice models (n = 5 for each group). As shown in Figure 4A, DCNP@PB failed to detect 4T1 tumor even at 12 h after injection until the tumor received an intratumoral injection of NaHS. In contrast, the HCT116 tumor region was clearly illuminated with a strong NIR-II luminescence at 4 h post-injection with DCNP@PB. However, the pretreatment of HCT116 tumor with AOAA significantly retarded the imaging process of DCNP@PB, reinforcing the key role of endogenous H_2_S in DCNP@PB activation. These results were supported by the quantitative analysis in Figure 4B, which showed that the DCNP@PB yielded a higher luminescence contrast, defined as a tumor-to-background ratio (TBR), in the HCT116 tumor than in the 4T1 tumor, consistent with *in vitro* results above.

To better investigate the performance of DCNP@PB for imaging of colorectal tumor, H_2_S-activatable DCNP@PB probe (OFF-ON) and non-activatable probe without PB shell (NdNP@PAA-mPEG), which was used as an Always-ON control probe were separately injected into HCT116 tumor-bearing mice via tail vein (n = 5 for each group). Compared with the whole-body distribution of NdNP@PAA-mPEG, clearly delineated tumor with a bright luminescence could be observed at 2 h post-injection with DCNP@PB (Figure 4C). Meanwhile, the signal was barely visible over non-tumor areas, indicating an exceptional ability of DCNP@PB to distinguish the tumor from normal tissue with high sensitivity and specificity. Further quantitative analysis clearly demonstrated a much higher contrast of DCNP@PB in the tumor than NdNP@PAA-mPEG (Figure 4D). The maximum contrast was reached in the DCNP@PB group at 24 h post-injection (TBR ~15.4) whereas the Always-ON probe had a minimal luminescence increase in the tumor over a 24 h period (TBR ~3.4). Such a marked colorectal tumor imaging ability could be attributed to the suitable H_2_S-response range of DCNP@PB, which helped to reduce the interference from other biomolecules* in vivo*, thereby ensuring desirable specificity toward H_2_S in colorectal tumor.

### NIR-II luminescence-guided surgery by DCNP@PB

The great tumor recognition capability of DCNP@PB inspired us to further examine the feasibility of DCNP@PB for NIR-II luminescence-guided surgery (Supplementary Video). DCNP@PB was injected intravenously into HCT116 tumor-bearing mice 12-24 h before surgery. After resection of the tumor under the guidance of DCNP@PB illumination, there was no detectable signals can be observed in the tumor bed, indicating that the tumor was excised completely (Figure S6A). Consistent with *in vivo* imaging result, DCNP@PB generated remarkable TBR (more than 10, Figure S6B). Meanwhile, e*x vivo* imaging of the tumor and major organs collected from mice that were sacrificed post-injection was performed to examine the biodistribution of the DCNP@PB. As shown in Figure S6C and D, DCNP@PB exhibited higher NIR-II signals in the tumor over normal organs, which was probably due to specific activation of DCNP@PB by H_2_S in the tumor region. As a result, the background signals can be effectively suppressed, giving rise to enhanced contrast for specific tumor visualization. All these results strongly supported the ability of DCNP@PB to specifically image the tumor rather than the normal tissue. Additionally, multiple physiological indicators in the blood biochemistry suggested good biocompatibility of DCNP@PB (Figure S7).

### Specific imaging of tumor tissues in patients with CRC

**Figure 5:**
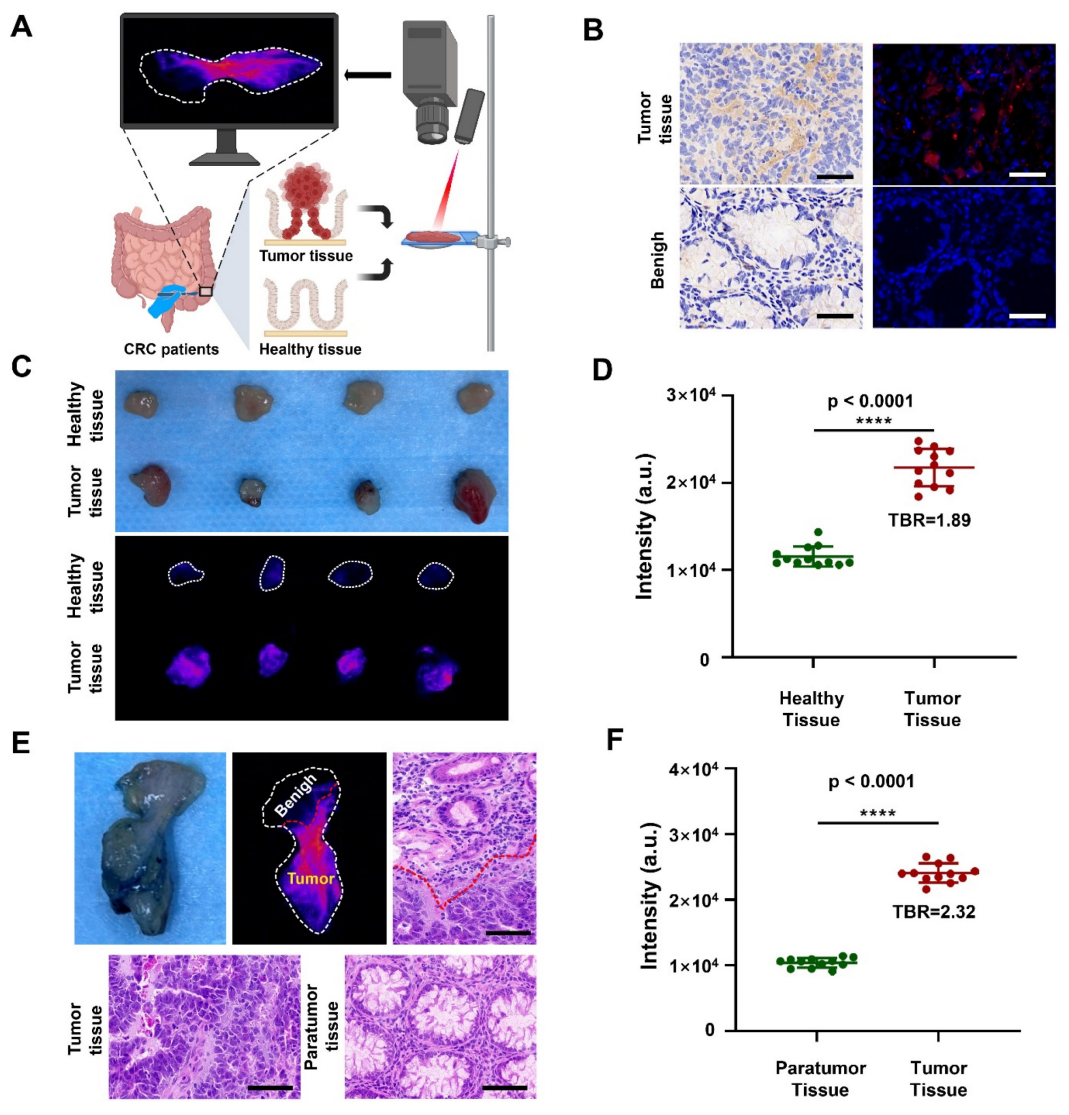
NIR-II imaging of tissues taken from the patients. (A) Schematic illustrations of NIR-II imaging of tumor tissues and healthy tissues taken from the patients. (B) Immunohistochemical staining (left) and immumofluorescence staining (right) of CBS in tumor tissues (upper) and benign (lower) taken from the patients. (CBS is red, scale bar: 50 μm) (C) Photo (upper) and NIR-II imaging (lower) of healthy tissues and tumor tissues taken from the patients. (D) Quantification of luminescence signals of healthy tissues and tumor tissues taken from the patients in (C). (E) Photo, NIR-II imaging and d H&E staining of tissue where both tumor and paratumor tissue of colon tissues taken from the patients. (scale bar: 100 μm) (F) Quantification of luminescence signals of paratumor tissue and tumor tissue taken from the patients in (E).

Having demonstrated the remarkable capability of DCNP@PB to detect the tumor in mouse models, we further explored its feasibility for precise discrimination between cancerous and normal human colorectal tissues (Figure 5A). Before that, we first measured the expression of CBS in tumor tissue of CRC patients. As shown in Figure 5B, immunohistochemical staining and immunofluorescence staining validated that CBS were highly expressed in tumor tissues of CRC patients, but hardly observed in benign tissues, indicating that the H_2_S level was indeed higher in human tumor than in normal tissues. These results encouraged us to employ DCNP@PB to image the resected human specimens during surgery, which were assessed by pathologic evaluation. As shown in Figure 5C and D, the tumor tissues were illuminated by DCNP@PB with high TBR (1.89), while adjacent healthy tissues only showed very weak signals. H&E staining confirmed that the NIR-II signals were highly tumor specific, indicating the effectiveness of DCNP@PB as a promising option for fast and precise tumor identification and histologic evaluation (Figure S8). It is noteworthy that our DCNP@PB excelled in delineating the tumor margin, validating the reliability of DCNP@PB to differentiating between malignant and benign lesions (Figure 5E and Figure S9). Such accurate tumor recognition of DCNP@PB could probably be attributed to its tumor-specific H_2_S responsiveness, which effectively reduced nonspecific activation by other sulfur-containing substances in complicated physiological environment.

## Conclusions

In summary, we successfully developed a H_2_S-activatable NIR-II probe DCNP@PB for highly specific imaging of CRC *in vivo*. By exploiting a novel PAA-mediated strategy, a homogeneous PB outer shell could be uniformly coated on the DCNP surface to ensure effective IFE and FRET between DCNPs and PB. As a result, luminescence of DCNP@PB can be efficiently switched from OFF to ON state in response to H_2_S. In the absence of H_2_S, DCNP@PB exhibited good stability and very weak emission. Upon arriving in the H_2_S-rich colorectal tumor, however, the luminescence of DCNP@PB at 1058 nm was recovered rapidly to illuminate the tumor. Notably, H_2_S-responsive range of DCNP@PB fell right into the H_2_S concentration range at the tumor site, which greatly reduced interference from other sulfur-containing substances in complicated physiological environment. Benefiting from such accurate responsiveness, DCNP@PB can be specifically activated by H_2_S in colorectal tumor rather than in normal organs/tissues even when DCNP@PB was intravenously administrated *in vivo*. Compared to the non-activatable probe, the H_2_S-activatable DCNP@PB probe can afford much higher TBR in tumor regions (16 vs 3), making DCNP@PB ideal candidate for real-time CRC imaging and image-guided surgery. Notably, DCNP@PB could not only possess the superior capability of DCNP@PB to detect the tumor in mouse models, but also ensure the precise discrimination between cancerous and normal human colorectal tissues. This work presents not only a promising example of H_2_S-activated NIR-II optical probe that could be intravenously injected for *in vivo* applications, but also an effective strategy to design the activatable probes for precise tumor imaging and fast histologic evaluation.

## Supplementary Material

Supplementary figures.

Supplementary video.
