# Supplementary material for: A H2S-activated NIR-II imaging probe for precise diagnosis and pathological evaluation of colorectal tumor
Source: Theranostics. 2025 Jan 1;15(1):189–201. doi: 10.7150/thno.103999 (PMC11667233; doi:10.7150/thno.103999)
Supplement: Supplementary file 1 — Supplementary figures. [file thnov15p0189s1.pdf]

Supporting Information

A H_2S-activated NIR-II imaging probe for precise diagnosis and pathological evaluation of colorectal tumor

Yu Ji[1,‡], Qinxian Huang[3,‡], Qian Jia[1,2✉], Haohao Yan[1], Yajing Chi[1], Yuanyuan Jia[1], Chaoqiang Qiao[1], Yanbin Feng[1], Zuo Yang[1], Ruili Zhang[1✉], Zhongliang Wang[1✉]

1. Lab of Molecular Imaging and Translational Medicine (MITM), Engineering Research Center of Molecular and Neuro Imaging, Ministry of Education, School of Life Science and Technology, Xidian University & International Joint Research Center for Advanced Medical Imaging and Intelligent Diagnosis and Treatment, Xi'an, Shaanxi, 710126, P. R. China.
2. Guangzhou Institute of Technology, Xidian University, Guangzhou, GuangDong, 510000, P. R. China.
3. Department of Neurogastroenterology and Pelvic Floor Surgery, Hospital of Gastroenterology, Xi'an International Medical Center Hospital, Xi'an, Shaanxi, 710100, P. R. China.

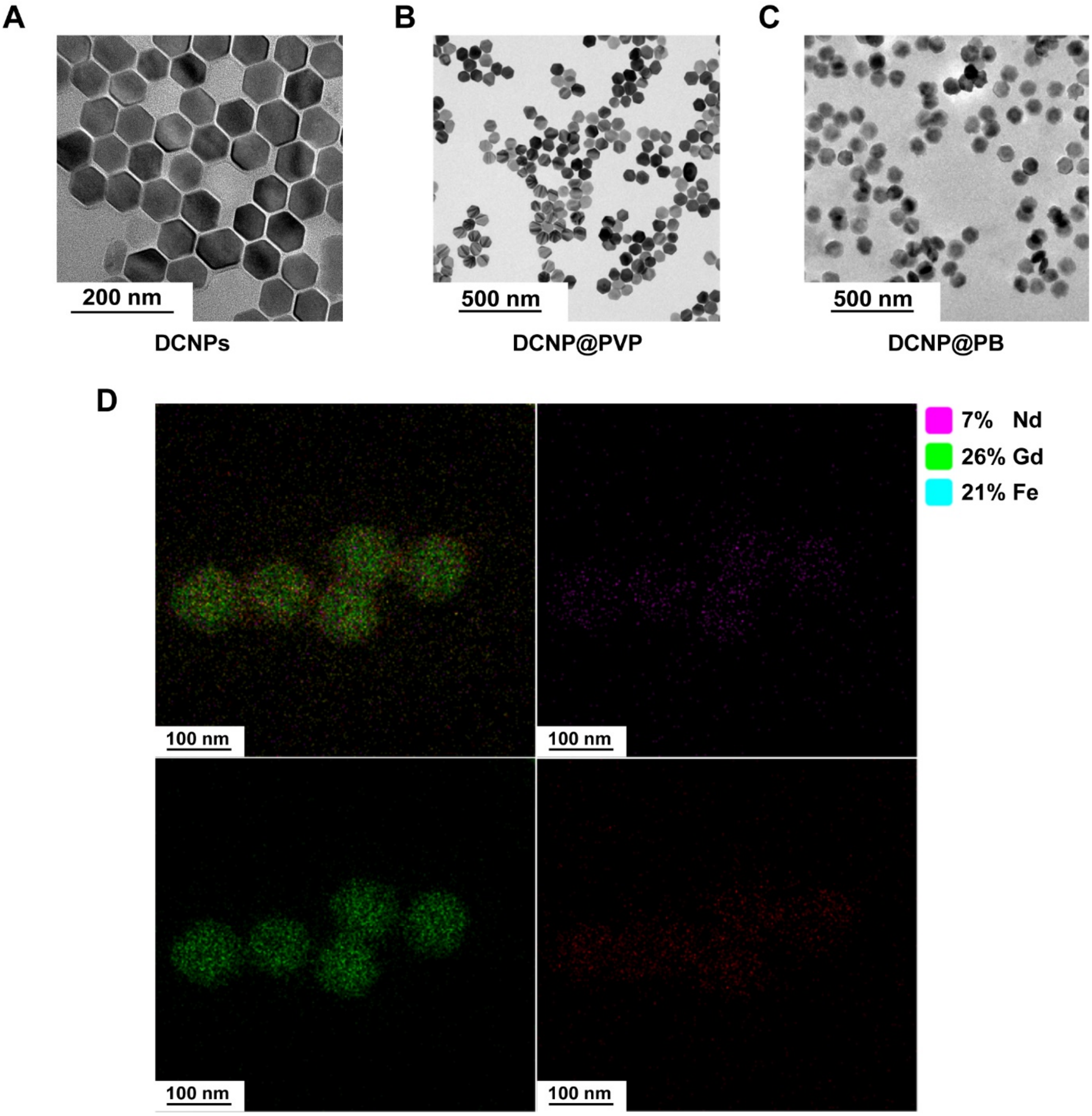

Figure S1. TEM images of DCNPs (A), DCNP@PVP (B) and DCNP@PB (C). (D) Elemental mapping analysis of DCNP@PB.

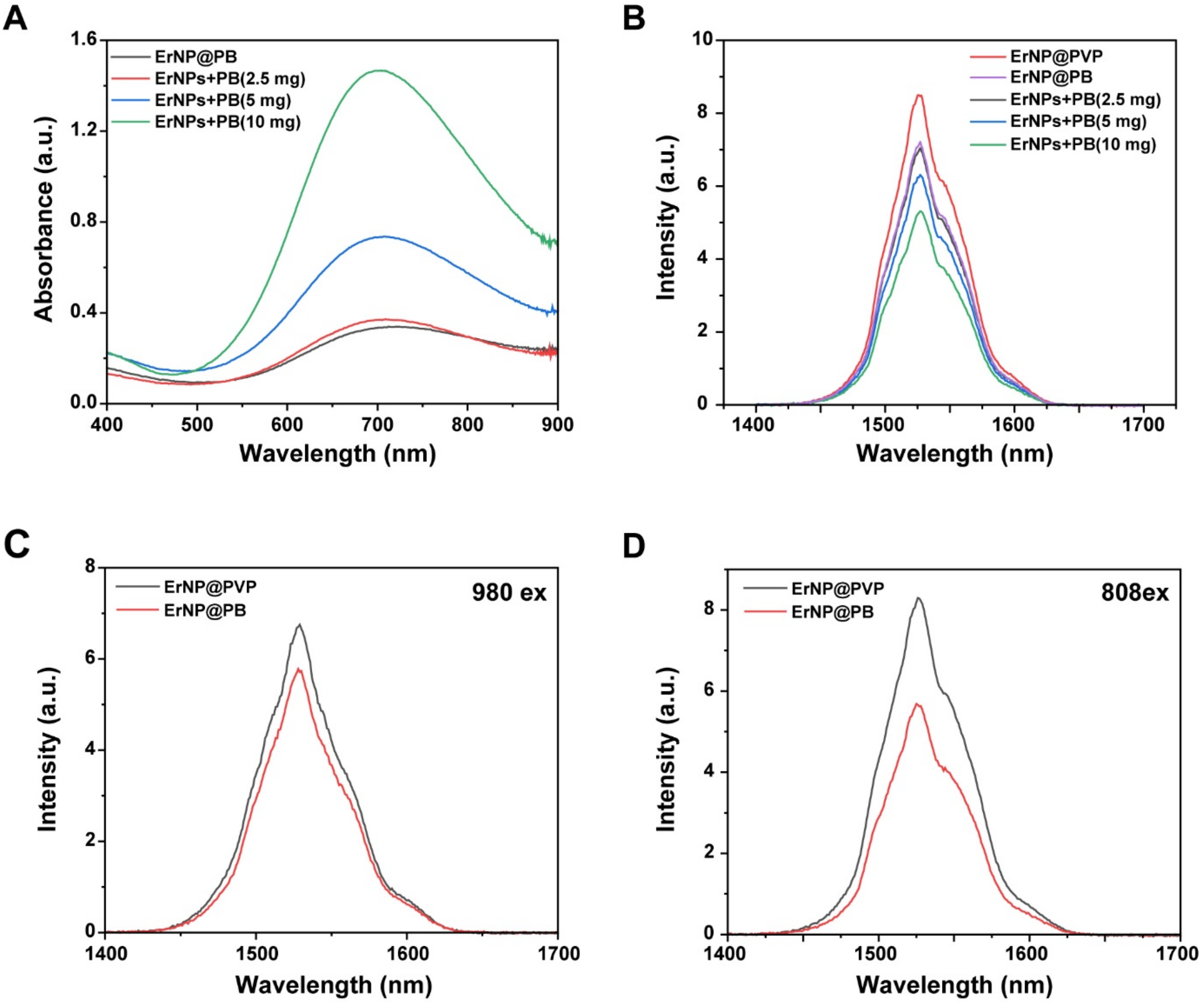

Figure S2. (A) Absorption spectrum of ErNP@PB and ErNP@PVP in PB solutions (2.5 mg, 5 mg,10 mg). (B) NIR-II emission spectrum of ErNP@PVP, ErNP@PB and ErNP@PVP in PB solutions (2.5 mg, 5 mg,10 mg). NIR-II emission spectra of ErNP@PVP and ErNP@PB excited by (C) 980 nm laser and (D) 808 nm laser.

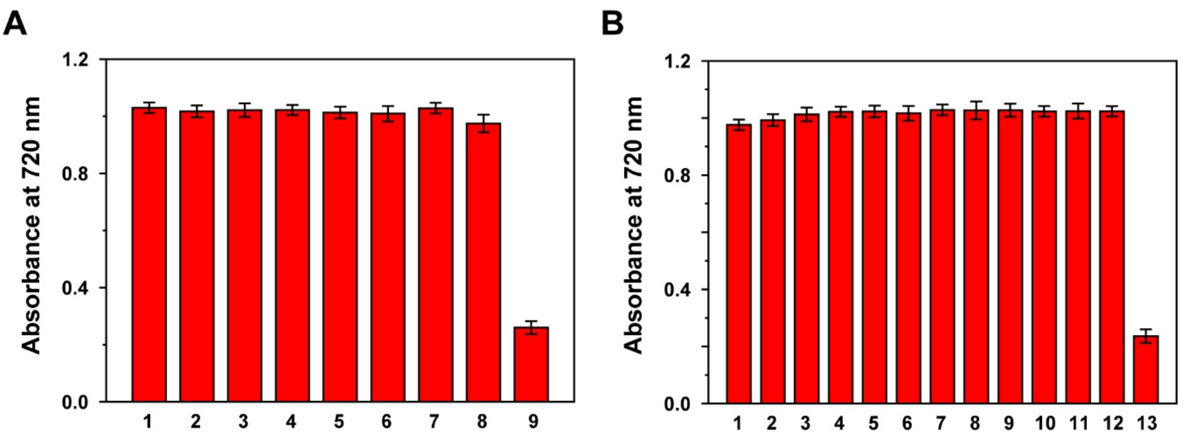

Figure S3. Specificity of DCNP@PB. (A) Statistical results of absorbance at 720 nm of DCNP@PB in the presence of H_2S or other active substances. (1. free 2. GSH 3. Cys 4. Glu 5. SO_3^{2-} 6. H_2O_2 7. VC 8. PO_4^{3-} 9. H_2S). (B) Statistical results of absorbance at 720 nm of DCNP@PB in the presence of H_2S or other active substances. (1. HClO 2. $ONOO^-$ 3. NH_4^+ 4. Cl^- 5. BSA 6. Dopamine 7. Na^+ 8. K^+ 9. Ca^{2+} 10. Mn^{2+} 11. Mg^{2+} 12. Zn^{2+} 13. H_2S).

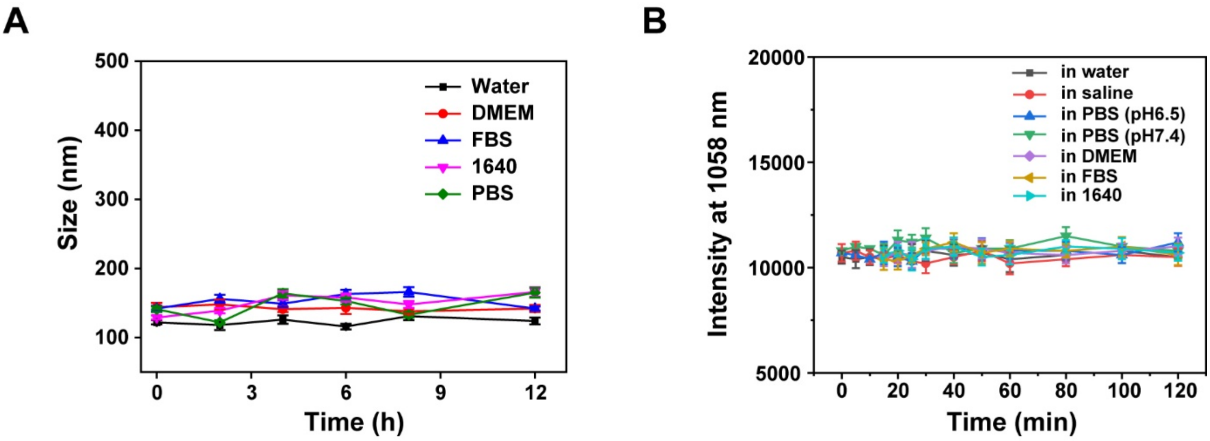

Figure S4. Size and luminescence stability of DCNP@PB in different solutions. (A) Size changes of DCNP@PB in water, PBS, DMEM, FBS, 1640 in 12 h. (B) Intensity at 1058 nm changes of DCNP@PB in water, saline, PBS (pH 6.5 and 7.4), DMEM, FBS and 1640.

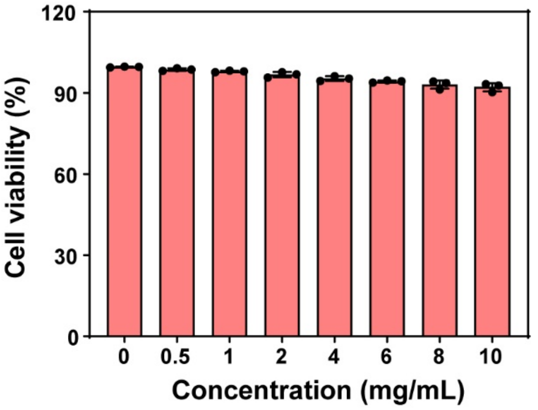

Figure S5. The MTT assay of HCT116 cells after treated with DCNP@PB at different concentrations for 24 h.

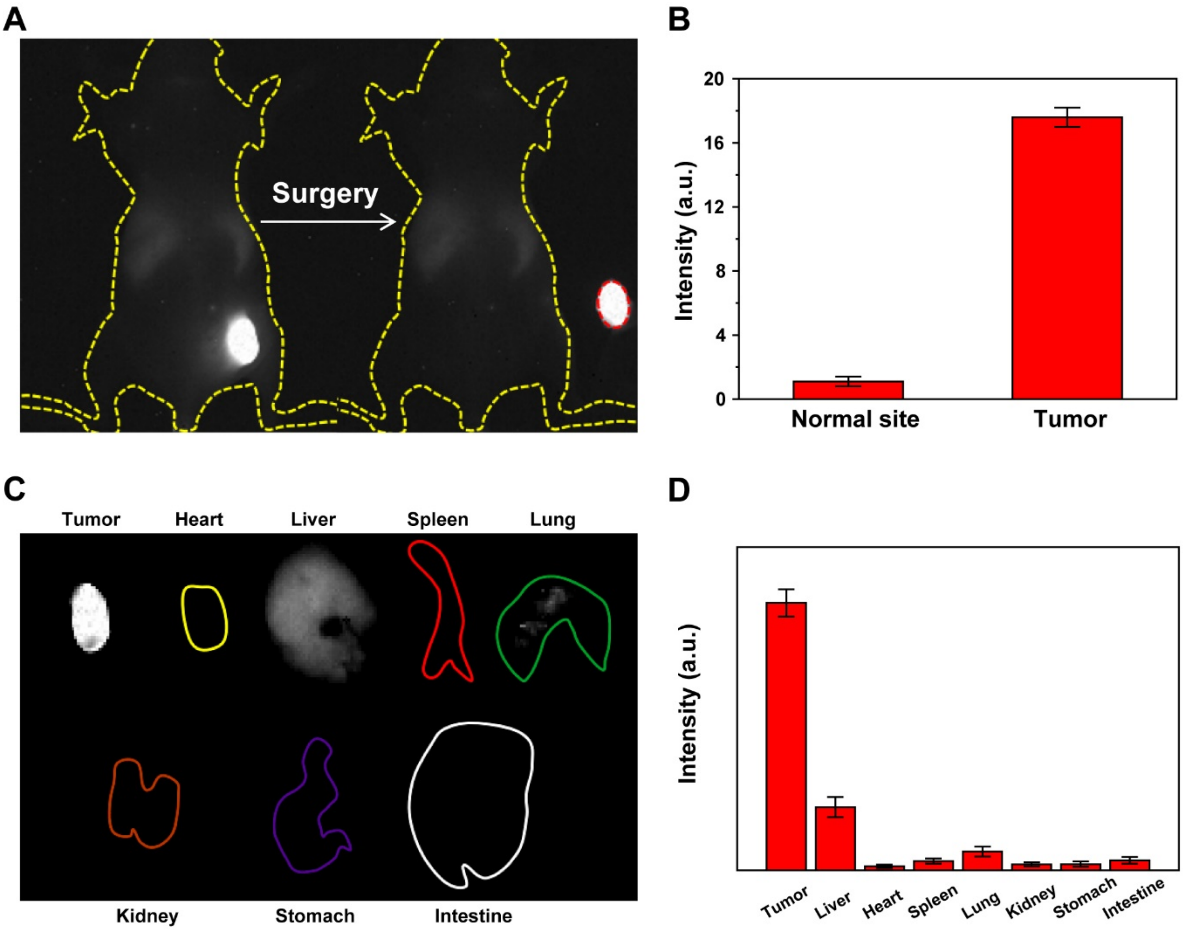

Figure S6. NIR-II luminescence-guided surgery by DCNP@PB. (A) NIR-II imaging of surgery for removing HCT116 tumor in mouse. (B) Statistical results of luminescence intensity of tumor and normal site during the surgery. (C) NIR-II imaging and (D) corresponding statistical results of luminescence intensity of tumor, heart, liver, spleen, lung, kidney, stomach, intestine.

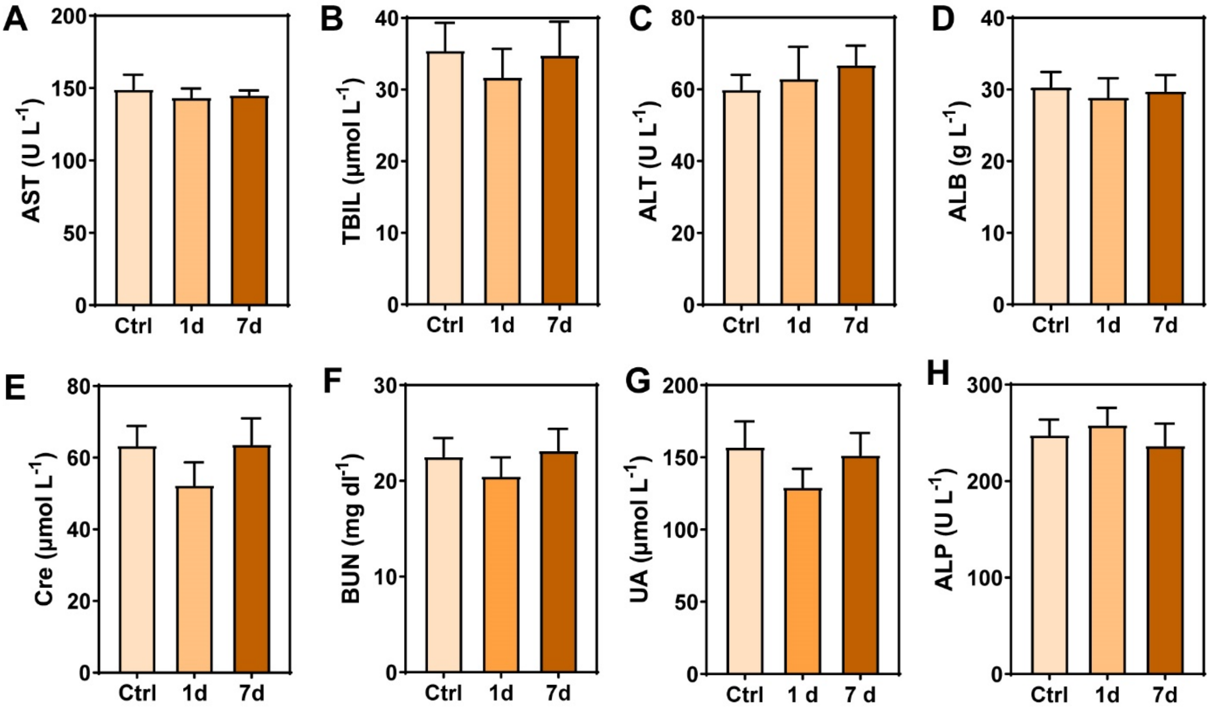

Figure S7. *In vivo* toxicity evaluation: blood biochemistry (A-H) of mice after intravenous injection of DCNP@PB or PBS (Ctrl) at different time. AST: aspartate aminotransferase, TBIL: total bilirubin, ALT: alanine aminotransferase, ALB: albumin, Cre: creatinine, BUN: blood urea nitrogen, UA: urea, ALP: alkaline phosphatase.

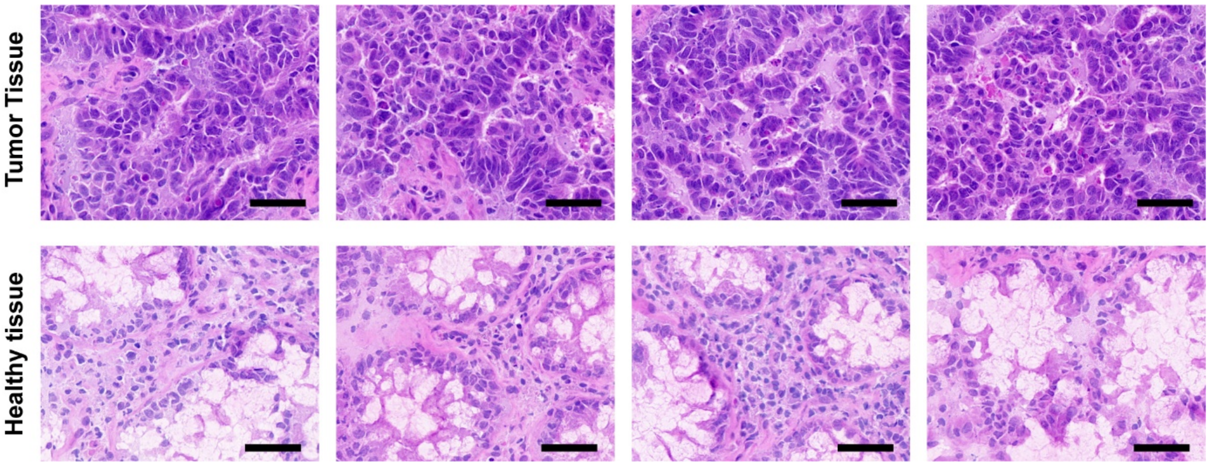

Figure S8. H&E staining of tumor tissues and healthy tissues from human *ex vivo* tissues (scale bar: 50 μm).

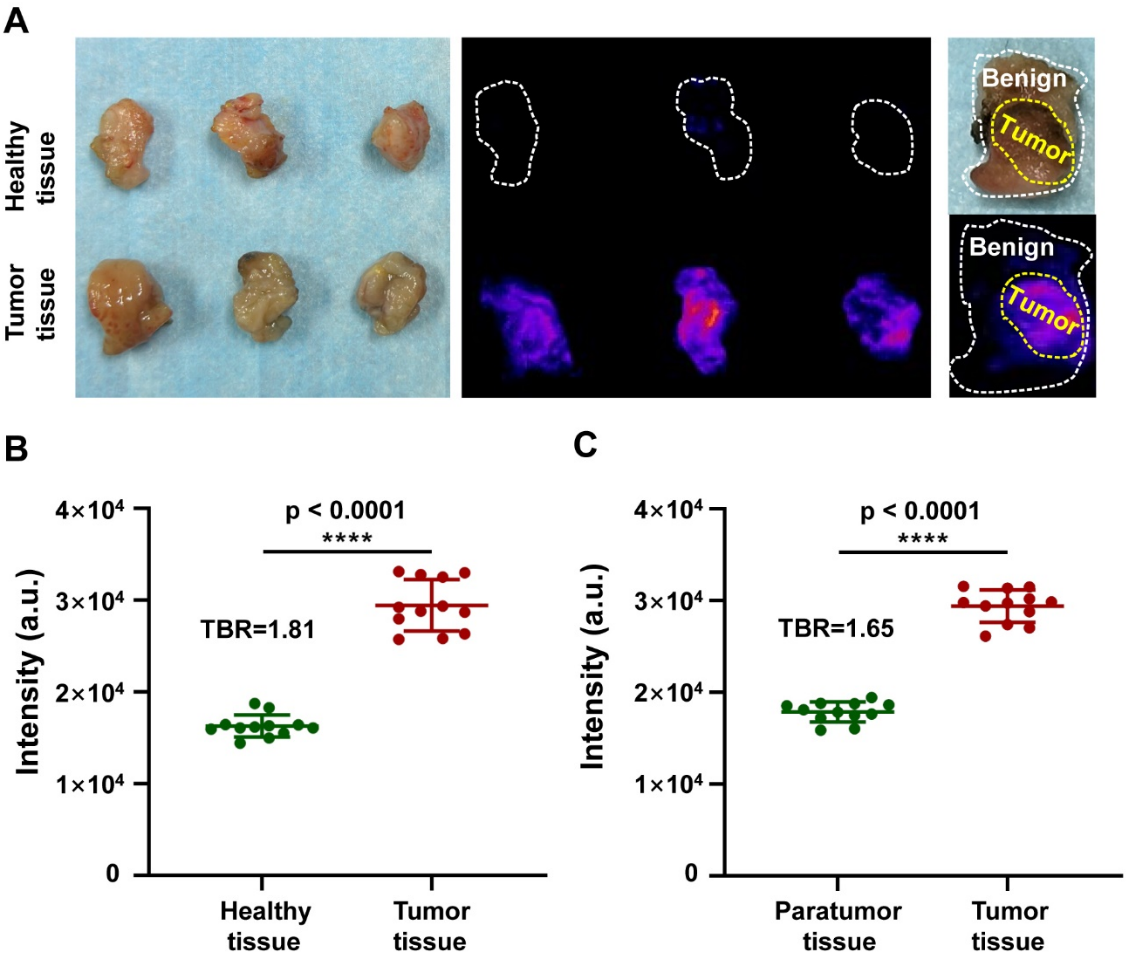

Figure S9. NIR-II Imaging of the batch-2 of tissues taken from the patients. (A) Photo and NIR-II imaging of healthy tissues and tumor tissues, tissue where both tumor and paratumor tissue of colon taken from the patients. Corresponding statistical luminescence luminescence signals of healthy tissues and tumor tissues (B) , tissue where both tumor and paratumor tissue (C).
